# Village and Country Town Concerts

**Published:** 1921-04-16

**Authors:** Walpole E. Sealy

**Affiliations:** (Hon. Organiser, Village and Country Town Concerts). Fonthill, East Grinstead, Sussex.


*To the Editor of* The Hospital.

### Village and Country Town Concerts.

*To the Editor of* The Hospital.

Sir,—I think it may interest some of your readers in the provinces to hear of this scheme, of which the enclosed circular gives a brief account. It would greatly help us in organising our tours to have a list of hospitals and institutions which might be glad of *free* concerts by any of our parties when touring in the various neighbourhoods. I should be glad to hear from the superintendents of any such hospitals, and have them by me for reference.

Since the circular was drawn up at Christmas we have given about 170 more concerts, bringing the total up to somewhere over 400 in all, of which over thirty have been in hospitals and similar institutes in various parts of the country.—Yours faithfully, Walpole E. Sealy

(Hon. Organiser, Village and Country Town Concerts).
Fonthill, East Grinstead, Sussex.

[This interesting scheme is referred to on page 56.—Ed. "The Hospital."]

